# Abiraterone Rechallenge Based on Sequential Testing of Androgen Receptor Splice Variant 7 Expression in Circulating Tumor Cells: A Case Report

**DOI:** 10.3389/fonc.2020.00495

**Published:** 2020-04-08

**Authors:** Naoya Nagaya, Mayuko Kanayama, Masayoshi Nagata, Shigeo Horie

**Affiliations:** Department of Urology, Juntendo University Graduate School of Medicine, Bunkyo, Japan

**Keywords:** abitaterone, androgen receptor splice variant 7, castration-resistant prostate cancer, circulating tumor cells, rechallenge

## Abstract

Serial analysis of circulating tumor cells (CTCs) such as androgen receptor splice variant 7 is useful in selecting treatments for castration-resistant prostate cancer (CRPC). We report a case who had been positive for androgen receptor splice variant 7 in CTCs before docetaxel, and was subsequently treated with abiraterone rechallenge because of the negative conversion of androgen receptor splice variant 7 following docetaxel. Although, the rechallenge of anti-androgen agent based on CTCs analysis is expected to be an effective approach, it is yet to be reported. Thus, we chose the candidate for abiraterone rechallenge based on serial CTCs analyses by the AdnaTest. As a result, the patient responded to abiraterone that he once had developed resistance to. Our findings reinforce the utility of AR-V7 as a biomarker in the setting of post-chemo androgen-targeted-therapy rechallenge.

## Background

Most metastatic prostate cancers are treated with androgen deprivation therapy (ADT) at the outset. Nevertheless, most of them progress to acquire resistance to the primary ADT, which is a state called castration-resistant prostate cancer (CRPC). Novel anti-androgen agents (enzalutamide, apalutamide, and darolutamide), CYP17A1 inhibitor that inhibit the production of testosterone (abiraterone), two taxane-based chemotherapies (docetaxel and cabazitaxel) and radium-223 have been approved by the US Food and Drug Administration for CRPC treatments (1–3).

Despite a variety of treatment options available for CRPC, there is no predictive biomarker used for treatment selection. Instead, clinicians decide on a course of treatment based on the several prospective randomized controlled phase 3 trials. For instance, most prostate cancer experts have consensus that asymptomatic men with metastatic CRPC should receive abiraterone or enzalutamide as the first-line treatment (4).

However, from the perspective of personalized medicine, we should choose treatments based on the genomic profiles of CRPC. Since genomic analysis of biopsy samples from metastatic lesions is not practical, liquid biopsy such as circulating tumor cells (CTCs) and circulating tumor DNA are drawing attention in recent years. In terms of CTCs, Antonarakis's study propounded testing androgen receptor splice variant 7 (AR-V7) expression in CTCs (5, 6). In their study, AR-V7-negative cohort showed a better prostate-specific antigen (PSA) response to novel anti-androgen agents than AR-V7-positive cohort. Furthermore, serial testing of AR-V7 in CTCs can be useful in selecting treatments for CRPC. However, this CTC analysis is yet to be acknowledged as the standard companion diagnosis. To further verify the utility of CTC analysis, prospective studies are currently underway (6).

**Figure 1:**
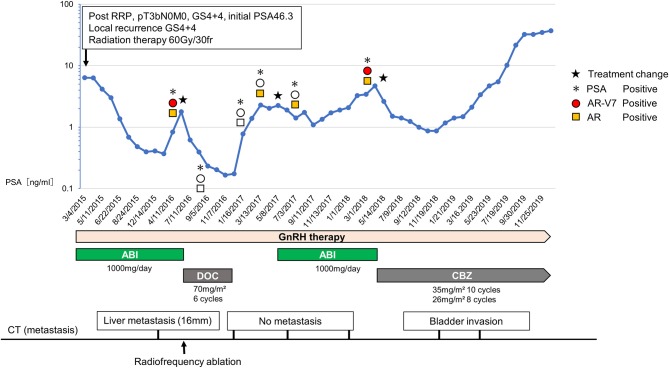
Changes of the serum PSA, treatment course, metastasis lesion, and the status of CTCs. The upper graph shows the changes of serum PSA and CTC status including AR-V7 expression. The treatment course is showed in the middle and the status of metastasis lesion is shown in the bottom. During the first-round abiraterone administration, when the serum PSA level rose twice, we analyzed AR-V7 expression in CTCs. Due to positive AR-V7 expression, we chose docetaxel as the second-line CRPC treatment. Because of rising PSA level during a drug holiday after docetaxel, we rechallenged abiraterone based on the negative conversion of AR-V7 expression, which resulted in PSA decline. When PSA rose to 4.00 ng/dl 10 months after abiraterone rechallenge, the expression of AR-V7 was converted to positive. Subsequently, cabazitaxel was started.

To this end, we performed a feasible bedside CTC testing for CRPC patients. Herein, we report a case of a CRPC patient who was successfully treated based on sequential CTC analysis (Figure 1).

This study was approved by the institutional review board of Juntendo hospital (admission number: 14-052), and all experiments were carried out in accordance with approved guidelines. Written informed consent was obtained from the patient for the publication of any potentially identifiable images or data included in this article.

## Case presentation

In November 2007, a 62-year-old man was diagnosed as localized prostate cancer with no metastasis (cT3bN0M0, Gleason score 4 + 4, initial PSA 46.3 ng/dl) and underwent radical prostatectomy. In April 2008, he had a PSA recurrence (2.4 ng/dl) and the combination androgen blockade therapy (LH-RH agonist + bicalutamide 80 mg/day) was initiated. In March 2015, he presented a local recurrence, and the treatment was switched to abiraterone (1,000 mg/day) combined with LH-RH antagonist and prednisolone. In addition, radiation therapy (60 Gy/30 fr) was done against the local recurrence, which resulted in marked tumor disappearance. His disease was well-managed with abiraterone for 13 months. In June 2016, he showed PSA progression and a liver metastasis occurred. At this point, AR-V7 was positive by the AdnaTest (QIAGEN, Germany). Therefore, we selected docetaxel of every 4 week (70 mg/m^2^) and a total of six cycles was given. Radiofrequency ablation (RFA) was performed for a liver metastasis since it was a singular and small lesion (<30 mm), which is the indication for RFA. After the administration of docetaxel, PSA declined to 0.166 ng/dl. Furthermore, CTC analysis confirmed the negative conversion of AR-V7 in CTCs. Due to the favorable response to docetaxel, docetaxel treatment was suspended and only LH-RH antagonist was continued for the following 5 months as a drug holiday. Subsequently, when PSA rose to the pre-docetaxel level (2.29 ng/dl), we tested AR-V7 expression again. Resultantly, because AR-V7 still remained negative, we opted for abiraterone rechallenge based on the following discussion with patient. The attending physician explained to the patient that enzalutamide may cause adverse events, such as fatigue and anorexia. Because the patient had no adverse events during the administration of abiraterone, he wanted to resume abiraterone. Also, because he had experienced symptoms of alopecia and numbness that adversely affected his job at the time of docetaxel administration, he did not want to be treated with chemotherapy while at work.

As a result, 6 months after the abiraterone rechallenge, PSA value became lower (1.08 ng/dl) than the level before abiraterone rechallenge. In line with this, no other metastases were found. PSA elevation was not observed for 8 months. Afterward, when PSA rose to 4.00 ng/dl 10 months after abiraterone rechallenge, we analyzed AR-V7 expression in CTC again. CTC analysis showed that the expression of AR-V7 was converted to positive. Based on the CTC analysis, subsequent cabazitaxel was started. Although, PSA was well-controlled for 9 months after cabazitaxel administration, PSA rose continually after 10 cycles of cabazitaxel, and computed tomography image showed the emerge of the bladder invasion.

## Discussion and Conclusions

Here, we reported the case of a CRPC patient with liver metastases, who was treated with abiraterone rechallenge based on AR-V7 status in CTCs.

Nakazawa et al. (7) reported the dynamic transition of AR-V7 status in CTCs and its utility for treatment selections. Their study showed the results of detailed CTC profiles and treatment courses in 14 patients. In this study, there was one patient showing continuous AR-V7 positivity, who was treated with androgen-targeted therapy rechallenge after docetaxel. However, he did not benefit from this rechallenge. Also, there was another patient who benefited from the first time abiraterone after docetaxel, when CTCs' AR-V7 status changed from positive to negative following docetaxel. However, unlike our case, there was no patient who benefited from the androgen-targeted-therapy rechallenge following chemotherapies in Nakazawa's study.

The clinical outcome of treatment with abiraterone or enzalutamide for patients who progressed on abiraterone has been shown in COU-AA-302 trial. Most of these patients received chemotherapy before subsequent abiraterone or enzalutamide. PSA response (defined as a PSA reduction of at least 50% from baseline) was observed in 24 of 55 patients who received subsequent abiraterone and in 22 of 33 patients who received subsequent enzalutamide (8). Besides, the results of phase III clinical trial, CARD trial, showed that cabazitaxel significantly improved clinical outcomes compared with androgen-targeted therapy (abiraterone or enzalutamide) in patients with metastatic CRPC previously treated with docetaxel (9). Although, the benefit of post-chemotherapy abiraterone or enzalutamide rechallenge was limited to some patient, these results still suggested that some cancer regained abiraterone or enzalutamide sensitivity after chemotherapy. However, we need to accurately identify the candidate patient who potentially benefit from androgen-targeted-therapy rechallenge. Therefore, our case provides clinical and biological rationale for AR-V7-based patients selection for the androgen-targeted-therapy rechallenge following chemotherapies.

In our report, changes in AR-V7 expression appeared to be reflective of the treatment effect. For example, the negative conversion of AR-V7 occurred after docetaxel and RFA for liver metastasis, both of which could have eliminated AR-V7-positive CTCs. Then, the withdrawal of docetaxel possibly stimulated the expansion of AR-V7-negative cells expressing full-length AR, which might have resulted in regained susceptibility to abiraterone rechallenge. Although, PSA elevation was observed after the abiraterone rechallenge, this treatment was able to suppress the increase in PSA for the following 8 months and delay the initiation of subsequent cabazitaxel treatment. Furthermore, abiraterone was effective only when AR-V7 was negative. These findings reinforce the utility of AR-V7 as a biomarker in the setting of post-chemo androgen-targeted-therapy rechallenge.

On the other hand, we have a variety of issues we need to address in CTC research. Firstly, whether CTCs represent the entire tumor characteristics is questionable. Secondly, AR-V7 is not the only mechanism driving CRPC progression. The states of other mechanisms responsible for treatment resistance in CRPC such as dysregulated PI3K–AKT signaling, WNT signaling pathway and DNA repair defects need to be elucidated (10). Thirdly, not only the wild-type full-length AR and AR splice variants mediate AR-signaling in CRPC, but also gene amplification and gain-of-function mutations are reportedly implicated in the sustained AR signaling in CRPC (11). Further investigations and technical developments are required to clarify the underlying heterogeneity of CTCs and CRPC biology in CTCs.

To the best of our knowledge, this is the first reported case of regaining susceptibility to the post-chemo anti-androgen agent with the concurrent negative conversion of AR-V7. Our result suggests that the rechallenge of post-chemo AR-targeted therapy based on AR-V7 testing can be a good strategy in treating CRPC.

## Ethics Statement

Written informed consent was obtained from the patient for the publication of any potentially identifiable images or data included in this article.

## Author Contributions

All authors have significantly contributed to the study and are in agreement with the content of the manuscript. Each author's contribution is as follows: NN and MK performed the experiments. NN, MK, MN, and SH designed the study. NN and MK wrote the manuscript, and MN and SH revised it.

### Conflict of Interest

SH has research grants from Astellas and Sanofi Aventis, honorarium from Astellas, Takeda, Astrazeneca and Sanofi Aventis and Janssen. The remaining authors declare that the research was conducted in the absence of any commercial or financial relationships that could be construed as a potential conflict of interest.
